# Transmesenteric robot-assisted pyeloplasty for ureteropelvic junction obstruction in horseshoe kidney

**DOI:** 10.1590/S1677-5538.IBJU.2015.0315

**Published:** 2016

**Authors:** Aaron M. Potretzke, Anand Mohapatra, Jeffrey A. Larson, Brian M. Benway

**Affiliations:** 1 Washington University School of Medicine, Division of Urologic Surgery, St. Louis, MO, USA;; 2 Urology Academic Practice, Cedars-Sinai Medical Center, Los Angeles, CA, USA

## INTRODUCTION

Transmesenteric laparoscopic pyeloplasty has previously been reported in two pediatric patients. To our knowledge, there has yet to be a report of a robotic-assisted transmesenteric pyeloplasty. We sought to present a video display and step-by-step demonstration of this approach.

## CASE

The patient is a 28-year old female with an obstructed ureteropelvic junction (UPJ) of the left moiety of a horseshoe kidney. The Da Vinci S robotic platform was used. After transperitoneal access was obtained, a window in the mesentery was identified, and the renal pelvis was exposed. A dismembered, spatulated pyeloplasty was performed with transposition of the UPJ to a more dependent portion of the renal pelvis. A ureteral stent and closed surgical drain were placed.

## RESULTS

The case was completed without complications. Operative time was 207 minutes. Foley catheter and drain were removed on postoperative days 1 and 2, respectively. The patient was discharged home on postoperative day 2, and the ureteral stent was removed after 8 weeks. Her diuretic T1/2 improved from 39 to 16 minutes. The differential function of the left moiety improved from 31% to 42%.

## CONCLUSIONS

Robot-assisted transmesenteric pyeloplasty is safe and feasible for the management of UPJ obstruction in select patients with a horseshoe kidney. Future study of its use in a larger number of patients is necessary to define its role in this unique population.

