# Supplementary material for: Transmesenteric robot-assisted pyeloplasty for ureteropelvic junction obstruction in horseshoe kidney
Source: Int Braz J Urol. 2016 May-Jun;42(3):626–7. doi: 10.1590/S1677-5538.IBJU.2015.0315 (PMC4920586; doi:10.1590/S1677-5538.IBJU.2015.0315)
Supplement: Related texts

# Letter: EDITORIAL COMMENT

**Authors:** Lucas Wiegand

This is a well-done video that adds to the literature in the fledgling field of minimally-invasive reconstructive urology. This video should encourage urologists to counsel their patients that this procedure can be performed safely using minimally-invasive techniques with good outcomes. The difficulty with laparoscopic and/or robotic approaches to upper ureteric strictures is when direct visualization shows that the fibrosis extends beyond what was thought preoperatively. In this situation, the surgeon is faced with the choice of trying to reposition the patient for minimally-invasive psoas hitch/Boari flap, opening, or trying to obtain more length of ureter or bridge a gap that may cause undue tension. While the outcome here is excellent, proper patient selection is key.

*Lucas Wiegand, MD* *Assistant Professor* *University of South Florida Morsani College of Medicine* *Tampa, FL, USA* *E-mail: lwiegand@health.usf.edu*
